# Characterization of serum protein expression profiles in the early sarcopenia older adults with low grip strength: a cross-sectional study

**DOI:** 10.1186/s12891-022-05844-2

**Published:** 2022-10-03

**Authors:** Jingqiong Wu, Longjun Cao, Jiazhi Wang, Yizhao Wang, Huimin Hao, Liping Huang

**Affiliations:** 1 TianJin University of Sport, No.16 Donghai Road, West Tuanbo New Town, Jinghai District, Tianjin, 301617 PR China; 2 Guangxi Medical University, Nanning, 530021 Guangxi PR China; 3 Tianjin Huanhu Hospital, Tianjin, 300350 PR China

**Keywords:** Sarcopenia in older adults, Serum proteomics, Clinical diagnosis, Data-independent acquisition, Serum lipoprotein particle

## Abstract

**Background:**

Sarcopenia refers to the progressive loss of skeletal muscle mass and muscle function, which seriously threatens the quality of life of the older adults. Therefore, early diagnosis is urgently needed. This study aimed to explore the changes of serum protein profiles in sarcopenia patients through a cross-sectional study, and to provide the reference for clinical diagnosis.

**Methods:**

This study was a cross-sectional study carried out in the Tianjin institute of physical education teaching experiment training center from December 2019 to December 2020. Ten older adults were recruited, including 5 sarcopenia and 5 healthy older adults. After a detailed diagnostic evaluation, blood samples were collected to prepare serum for proteomic analysis using the HPLC System Easy nLC method. The differentially expressed proteins (DEPs) were screened by the limma package of R software (version 4.1.0).

**Results:**

A total of 114 DEPs were identified between the patients and healthy older adults, including 48 up-regulated proteins and 66 down-regulated proteins. The functional enrichment analysis showed that the 114 DEPs were significantly enriched in 153 GO terms, which mainly involved in low-density lipoprotein particle remodeling, and negative regulation of immune response,etc. The PPI network further suggested that the cholesteryl ester transfer protein and Apolipoprotein A2 could serve as biomarkers to facilitate diagnosis of sarcopenia.

**Conclusions:**

This study provided a serum proteomic profile of sarcopenia patients, and identified two proteins with diagnostic value, which might help to improve the diagnostic accuracy of sarcopenia.

**Supplementary Information:**

The online version contains supplementary material available at 10.1186/s12891-022-05844-2.

## Background

Sarcopenia is one of the biggest threats to the quality of life of older adults, characterized by loss of skeletal muscle mass and decreased muscle strength and function [1]. Risk factors for sarcopenia include age, gender, eating behavior, level of physical activity, and chronic diseases [2–4]. In fact, the incidence of sarcopenia varies greatly depending on the population surveyed, living conditions, and assessment tools and methods, hence its prevalence may vary widely. Recent studies have shown that the incidence of sarcopenia in the older adults was as high as 17% ~ 62% [5]. Therefore, sarcopenia will bring an increasing burden on health of older adults with an aging population.

Early screening and diagnosis can not only help us fully understand the occurrence of sarcopenia in the older adults, but also provide specific prevention and treatment approach. The European working group on sarcopenia on older people (EWGSOP) published diagnostic criteria for sarcopenia in 2010 (EWGSOP1) and revised it in 2018 (EWGSOP2), recommending poor muscle strength as a key feature of the disease and then using biompedance analysis to evaluate skeletal muscle index for further diagnosis [6]. Currently, the last update of the EWGSOP was in 2019 [7]. The diagnostic criteria were in the order of low muscle strength, low muscle quantity or quality, and low physical performance. If all of them were met, sarcopenia was considered severe [8]. The SARC-F (kown as a 5-item questionnaire, including 5 domains: strength, assistance with walking, rise from a chair, climbing stairs, and falls) was also considered as an important reference for diagnosis [9]. However, the assessments of physical activity could only indicate the severity of the disease [7]. Several tools available for the diagnosis of sarcopenia, such as magnetic resonance imaging and computed tomography, were limited by their high cost, complexity, and exposure to large amounts of radiation [10]. Therefore, we need to take a multi-aspect approach to develop new indicators to facilitate diagnosis. Currently, the serum proteomics has been widely used in the investigation for diagnostic markers of many diseases [11–13]. For example, Kimura Yayoi et al. used MS-based proteomic analysis to identify 1879 disease-related proteins, among which leucine-rich alpha-2- glycoprotein could serve as a biomarker for diagnosis of Kawasaki disease [14]. Therefore, systematic serum proteomics studies in sarcopenia patients will provide clues for the investigation of effective diagnostic biomarkers.

In present study, we aimed to identify the novel biomarker for diagnosis of sarcopenia patients via serum proteomics analysis, the underlying pathways were also revealed to clarify the pathogenesis, which may provide new diagnosis options for sarcopenia in the future.

## Subjects and methods

### Study design

This study was a cross-sectional study carried out in the Tianjin institute of physical education teaching experiment training center from December 2019 to December 2020. A total of ten older adults were recruited, including 5 patients and 5 healthy older adults. After a detailed diagnostic evaluation, blood samples were collected to prepare serum for proteomic analysis using the HPLC System Easy nLC method. Patient consent was obtained in this study, and the study was approved by the Institutional Ethics Committee.

This study adhered to the principle of randomization to control bias, and selected the study objects strictly according to the sampling design. The instruments and equipment were calibrated in advance to ensure the accuracy and reliability of the test results.

### Study objects

A total of 10 older adults were recruited according to the diagnostic process of the 2019 EWGSOP2 [7]. This study was conformed to the declaration of Helsinki and approved by the local ethics committee. The exclusion criteria were as follows:

1. Patients with motor dysfunction or recent exercise contraindications, such as osteoarthritis, severe chronic obstructive pulmonary disease, acute myocardial infarction, high-risk unstable angina, risk of thrombosis, etc.;
2. Patients with obvious mental illness or severe cognitive impairment (Briefly, we used a psychiatric assessment questionnaire such as have you had an uncontrollable tantrum recently? Never to serious);
3. Patients with severe cardiovascular and cerebrovascular diseases, such as cardiac surgery, atrial fibrillation, ventricular fibrillation, stroke, etc.;
4. Patients have taken drugs that affect body weight (such as metformin, acarbose, dapagliflozin, and empagliflozin);
5. Patients suffer from diseases that affect muscles, such as paralysis, muscular dystrophy, central nervous system diseases, etc.;
6. Other serious diseases: acute infection, rheumatic disease, severe liver, heart or kidney dysfunction.

### Patient selection criteria

All the patients were carefully evaluated including: handgrip strength test, bioelectrical impedance analysis [15], 6-minute walk test [16]. And then the skeletal muscle index (SMI) was calculated. SMI = ASM [kg]/height [Ht]^2^ [m] (ASM: appendicular skeletal muscle mass) [17]. Finally, the older adults were grouped according to the test results, and the criteria were as follows: (1) The healthy OAs group (N group, *n* = 5): age ≥ 60; handgrip strength: male ≥26 kg, female ≥18 kg; SMI: male > 7.0 kg/m^2^, female > 5.7 kg/m^2^. (2) The early sarcopenia OAs group (L group, *n* = 5): age ≥ 60; handgrip strength: male ≤26 kg, female ≤18 kg; SMI: male < 7.0 kg/m^2^, female < 5.7 kg/m^2^. *Serum sample collection.*

All older adults in two groups were collected 6–8 mL of blood intravenously under fasting condition, and the blood samples were placed in 37 °C for 30 min. Then centrifuged at 3000 g at 4 °C for 10 min [18], and collected the upper serum after confirming that there was no hemolysis and the supernatant was collected to store in − 80 °C refrigerator until mass spectrometry (MS) analysis.

### Proteomic analysis

The data-independent acquisition (DIA) was a newly developed MS technology. Compared with the traditional data-dependent acquisition (DDA) technology, it could fragment and detect all ions, and the information of all ions in the sample could be fully obtained [19]. In this study, DDA was used to construct a spectral library for subsequent DIA data analysis [20]. For proteomic analysis, the process mainly included the following steps: protein extraction, peptide enzymatic hydrolysis, chromatographic fractionation, DDA collection of LC-MS/MS data, DIA collection of LC-MS/MS data, construction of library, protein DIA data identification and quantitative analysis, differential expression proteins (DEPs) screening, functional annotation and pathway enrichment analysis, etc.

#### Serum sample preparation

For each serum sample, the serum protein concentration was determined by Bradford assay, then the serum protein was extracted with SDT lysate (4% SDS (161–0302, Bio-Rad), 150 mM TRIS-HCl (A6141, Sigma), 100 mM DTT (161–0404, Bio-Rad), pH 8), and an equal amount of protein from each sample was mixed as one pool sample to construct spectral library. In this study, we obtained 11 samples, including 5 serum samples of healthy older adults and 5 serum samples of patients, as well as one pool sample mixed with 5 serum samples of healthy older adults and 5 serum samples of patients. SDS-PAGE electrophoresis was used to evaluate the consistency of all samples. Enzymolysis was next performed on all samples according to the previously filter aided sample preparation (FASP) procedure [21]. The concentration of peptide was determined by OD280. All samples were mixed with 2 μg iRT (indexed retention time [22]) standard peptide, and DIA-MS was performed, respectively. The peptides in the pool sample were subjected to HpRP fractionation and all fractions were collected. Next, 2 μg peptide was transferred from each fraction and mixed with an appropriate amount of iRT standard peptide (volume proportion 1: 3), and then detected by DDA-MS for library construction.

#### MS method

All fractions for DDA library generation were analyzed by a Thermo Scientific Q Exactive HF X mass spectrometer connected to an Easy nLC 1200 chromatography system (Thermo Scientific). The peptide (1.5 μg) was first loaded onto an EASY-Spray TM C_18_ Trap column (Thermo Scientific, P/N 164946, 3 μm, 75 μm*2 cm), then separated on an EASY Spray TM C_18_ LC analytical column (Thermo Scientific, ES802, 2 μm, 75 μm*25 cm) with a linear gradient of buffer B (84% acetonitrile and 0.1% formic acid) at a flow rate of 250 nL/min over 120 min. MS detection method was positive ion, the scan range was 300–1800 m/z, resolution for MS1 scan was 60,000 at 200 m/z, target of AGC (automatic gain control) was 3e6, maximum IT was 25 ms, dynamic exclusion was 30.0 s. Each full MS-SIM scan followed 20 ddMS2 scans. Resolution for MS2 scan was 15,000, AGC target was 5e4, maximum IT was 25 ms and normalized collision energy was 30 eV.

The peptides from each sample were analysed by LC-MS/MS operating in the DIA mode. Each DIA cycle contained one full MS-SIM scan, and 30 DIA scans covered a mass range of 350–1800 m/z with the following settings: SIM full scan resolution was 120,000 at 200 m/z; AGC 3e6; maximum IT 50 ms; profile mode; DIA scans were set at a resolution of 15,000; AGC target 3e6; max IT auto; normalized collision energy was 30 eV. Runtime was 120 min with a linear gradient of buffer B (84% acetonitrile and 0.1% Formic acid) at a flow rate of 250 nL/min. QC samples (pool sample from equal aliquot of each sample in the experiment) were injected with DIA mode at the beginning of the MS study and after every 6 injections throughout the experiment, which was used to monitor the MS performance. Moreover, in DIA data, each chromatographic peak contained 5 data collection points to ensure that sufficient density data could be collected for accurate integral quantification of peptide chromatographic peaks.

#### Data analysis

DDA data was imported into Spectronaut™ 14.4.200727.47784 software to construct spectral library. The human_uniprot was used to download the database. The retrieval parameters are set as follows: enzyme: trypsin, Max Miss cleavage site: 1, fixed modification: carbamidomethyl, dynamic modification: oxidation and acetyl (Protein n-term), proteins identified in database searches must pass the filter parameter FDR < 1%. DIA data were processed by Spectronaut™ 14.4.200727.47784 software. Software parameter settings were as follows: retention time prediction type: dynamic iRT, interference on MS2 level correction: enabled, cross run normalization: enabled. All results must conform to the filter parameter Q-value-cutoff 0.01 (equivalent to FDR < 1%).

### Screen DEPs

The DEPs analysis was analysis by limma package of R software (version 4.1.0), and the DEPs were screened with the thresholds of |log_2_ FC| > 0.5 and *P*-value < 0.05.

### Functional enrichment analysis

The Database for Annotation, Visualization, and Integrated Discovery (DAVID) was used to perform the functional enrichment analysis. The *P*-value < 0.05 was considered as the threshold to select the significantly enriched Gene Ontology (GO) terms and Kyoto Encyclopedia of Genes and Genomes (KEGG) pathways.

### Protein-protein interaction (PPI) network construction

The STRING (https://string-db.org/) database was used to generate a PPI network of the DEPs [23], and use the combined score > 0.4 as the threshold to screen gene interactions. Cytohubba plug-in in Cytoscape (version3.6.1) was used to analyze the topology of PPI network to determine the core proteins in the module [24]. In the network, the nodes represented proteins and the edges represented protein-protein associations.

### Statistical analysis

All statistical analysis was completed using R software (version 4.1.0). *P*-value < 0.05 was used as the significance criterion.

## Results

### Quality control (QC) of protein quantitative

**Fig. 1:**
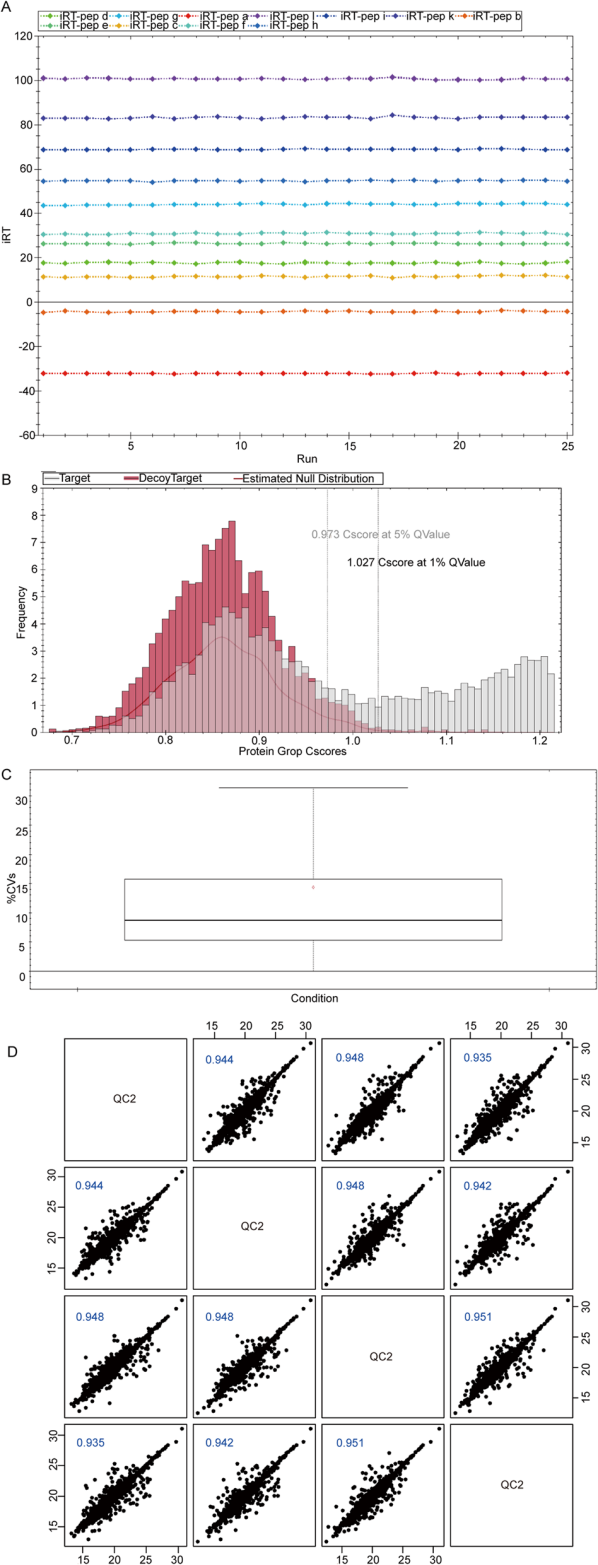
Quality control of protein quantitative in proteomic analysis. **A** The elution time of corrected peptide (iRT), vertical axis: retention time, horizontal axis: loading order. **B** FDR distribution map. Cscore: equivalent to protein reliability score, the higher the score, the greater the reliability. Horizontal axis: Cscore value of protein; Vertical axis: the number of proteins under a certain Cscore score. Black dotted line: 1% Q-value (equivalent to 1% FDR) standard line, the higher the Cscore at the standard line, and the higher the reliability. **C** Box diagram of CV value distribution of QC, the median CV value of QC samples was 8.7%. **D** Correlation analysis of QC samples. Log values of intensity values were marked on the horizontal axis and vertical axis respectively, and correlation coefficients greater than 0.9 indicating a high correlation

Firstly, the results of internal standard modified peptide (iRT) detection showed that its retention time was generally stable (Fig. 1A), indicating that the detection system was reliable. The QC based on target-decoy search strategy was used in DIA data analysis, and we found that results with FDR threshold value as 0.01 have higher reliability (Fig. 1B). Moreover, we inserted a QC sample into the sample queue at an interval of a certain number of samples, and evaluated the data consistency of the inserted QC samples throughout the experiment. Intra-group coefficient of variation (CV) and pearson correlation analysis were used to evaluate the consistency of QC samples. The lower of CV values and the closer the correlation was to 1, the more stable of experimental system. As the results, the median CV was 8.7% (Fig. 1C), and the pairwise correlation coefficient of all QC samples was greater than 0.9 (Fig. 1D). These results indicated that the experimental system was stable and reliable.

### Identification of sarcopenia-related proteins

**Fig. 2:**
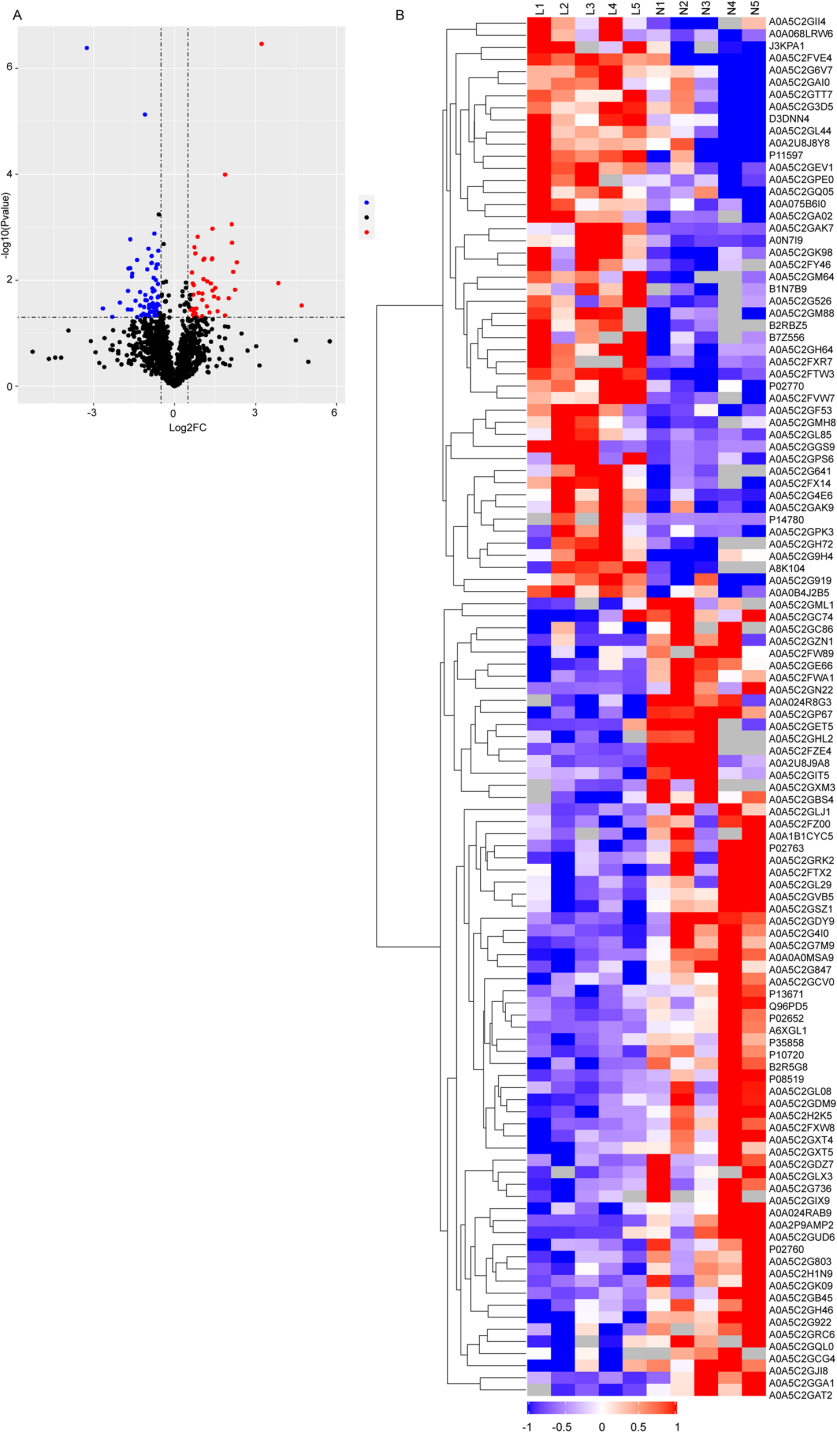
Identification of DEPs between the healthy group and the early sarcopenia group. **A** Volcano diagram of DEPs, the horizontal axis was logarithmic differential expression multiple (Log_2_FC), the vertical axis was -Log10 (FDR), blue dots were down-regulated proteins, and red dots were up-regulated proteins. **B** Heat map of DEPs. Horizontal axis: the samples (N: the healthy group, L: the early sarcopenia group). Vertical axis: the proteins. The red represented significantly up-regulated proteins, blue represented significantly down-regulated proteins

In this study, a total of 114 DEPs were identified as sarcopenia associated proteins by LC-MS/MS combined with DIA. Compared with the healthy group, there were 48 proteins were up-regulated and 66 proteins were down-regulated (Fig. 2A), and the DEPs showed significant differences between two groups (Fig. 2B).

**Fig. 3:**
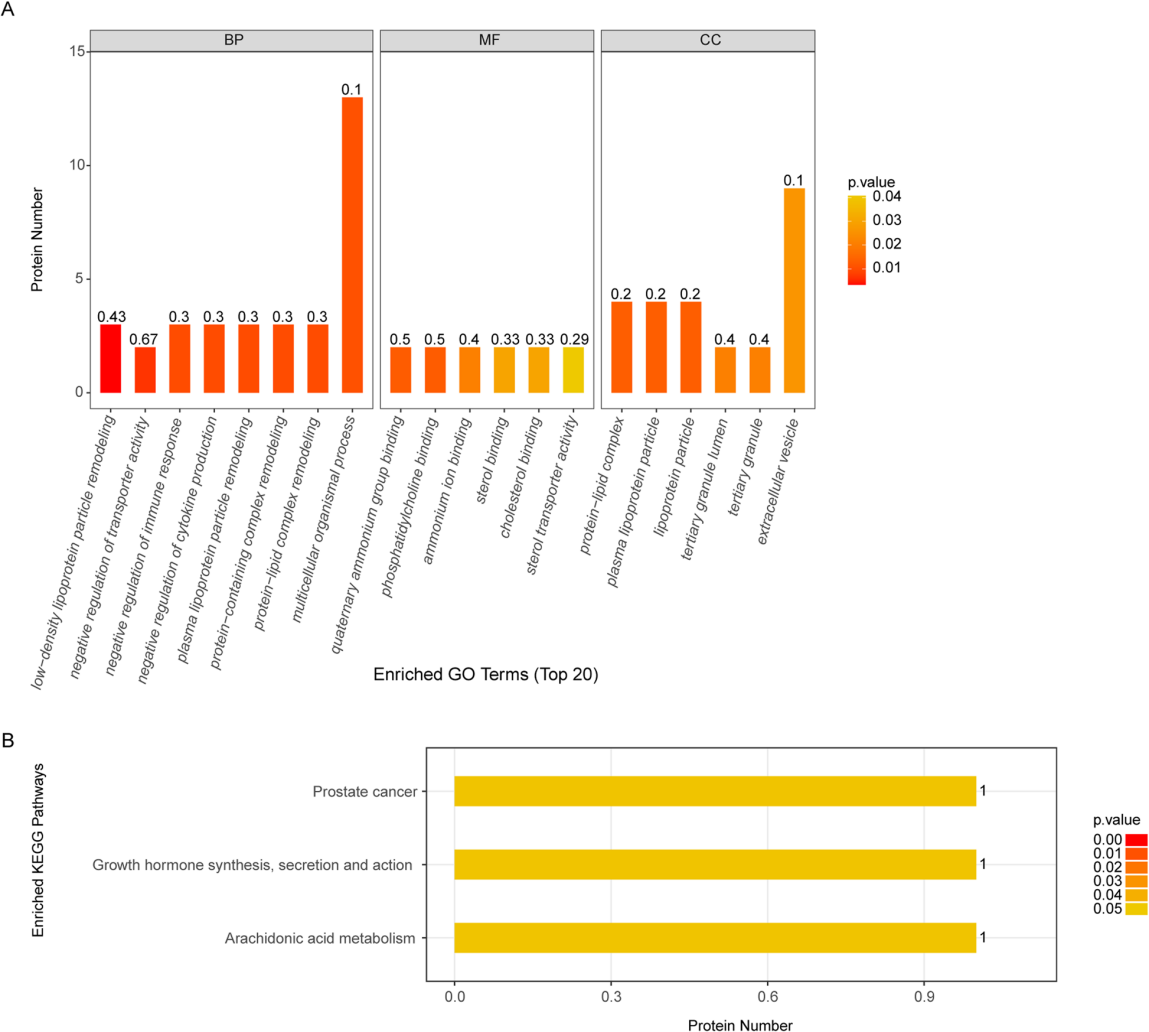
GO function and KEGG pathway enrichment analysis. **A** The significantly enriched GO terms. Vertical axis: the number of enriched genes, horizontal axis: the names of each GO terms. **B** Three KEGG pathways that were significantly enriched. Vertical axis: the number of enriched genes, horizontal axis: the names of three KEGG Pathways

Furthermore, the GO-biological process suggested that the 114 DEPs were significantly enriched in 153 GO terms (Table S1). The top 20 significant results of the enrichment analysis for biological process (BP), cellular component (CC) and molecular function (MF) were shown in Fig. 3A. As for BP, the DEPs were mainly enriched in the low-density lipoprotein particle remodeling, negative regulation of transporter activity, negative regulation of immune response, and negative regulation of cytokine production, etc. In the CC group, the DEPs were mainly enriched in the protein-lipid complex, plasma lipoprotein particle, and lipoprotein particle, etc. In the MF classification, the DEPs were mainly enriched in quaternary ammonium group binding, phosphatidylcholine binding, and ammonium ion binding, etc. The KEGG pathway enrichment analysis showed that the DEPs were mainly involved in prostate cancer, growth hormone synthesis, secretion and action, and arachidonic acid metabolism (Fig. 3B, Table S1)(The pathways were obtained baising on KEGG [25–27]). We noticed that most of these biological processes were involved in serum lipoprotein metabolism and transport [28], suggesting that patients with sarcopenia might develop dyslipidemia, which was consistent with the study of Gong et al. [29].

### The PPI network based on DEPs

**Fig. 4:**
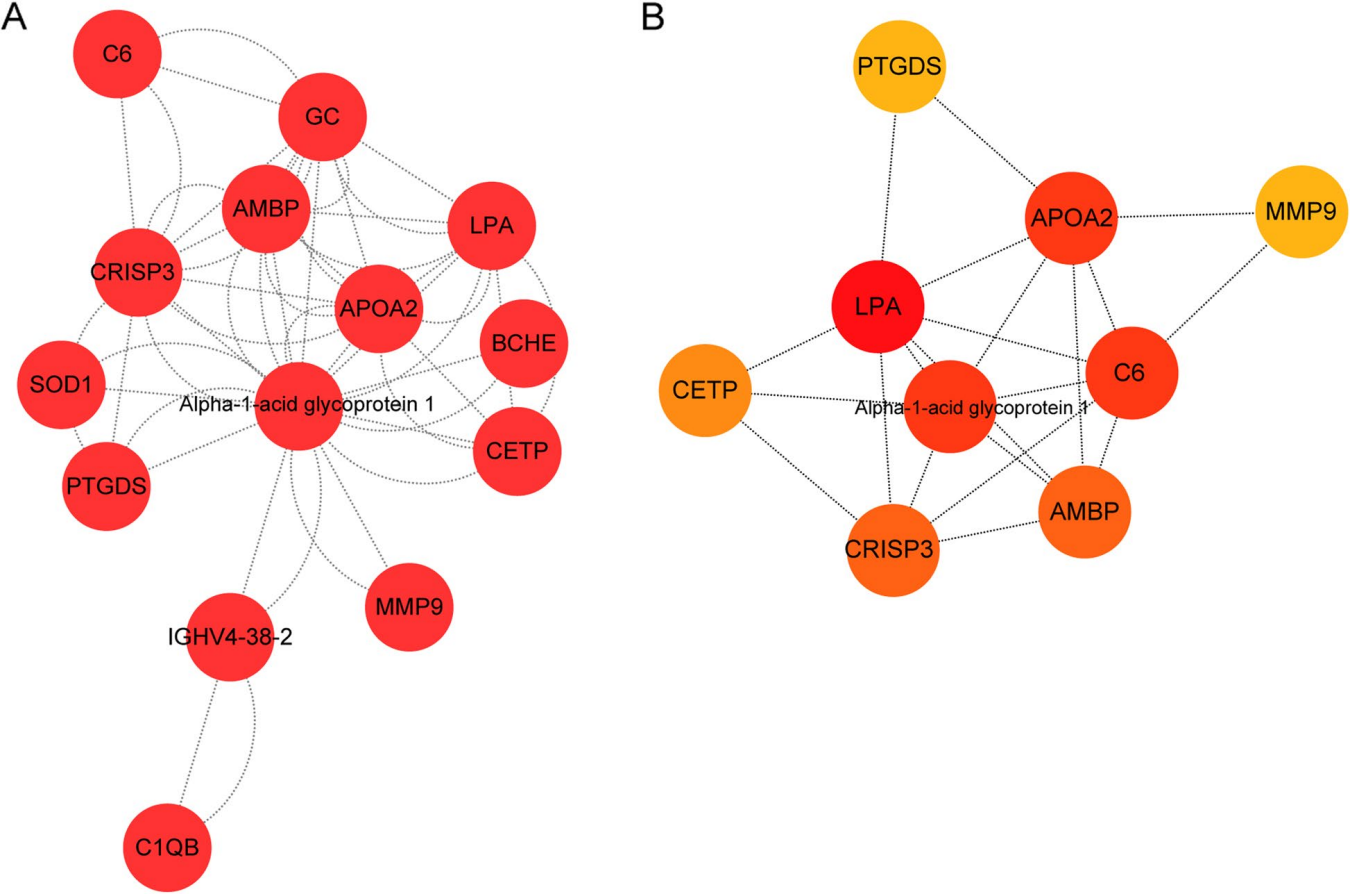
PPI networks revealed the key proteins. **A** PPI network diagram, each dot in the network represented a protein, and the more segments connected with the dot, the more important this protein was in the network structure; **B** The top 9 proteins with higher degree in PPI network screened by MNC algorithm. The darker the red color, the higher the degree

The PPI network revealed the potential connection between proteins [30]. In present study, we constructed a PPI network based on 114 DEPs by STRING database. As the results, the network contained the 14 nodes and 52 relationship pairs (Fig. 4A). Then the MNC algorithm in Cytohubba plug-in was used to calculated the top 9 genes with the highest connectivity in the network including prostaglandin D2 synthase (PTGDS), cysteine-rich secretory protein 3 (CRISP3), apolipoprotein A2 (APOA2), α-1-microglobulin/bikunin precursor (AMBP), Alpha-1-acid glycoprotein 1, lysophosphatidic acid (LPA), cholesteryl ester transfer protein (CETP), C6, and matrix metalloproteinase-9 (MMP-9) (Fig. 4B). As shown in the Fig. 3B, a node represented a gene and an edge represented the interrelationship between two connected proteins. These 9 genes correspond to 9 proteins, which were A0A024R8G3, J3KPA1, P02652, P02760, P02763, P08519, P11597, P13671, and P14780. We noticed that, compared with the healthy group, the expression levels of J3KPA1 (CRISP3), P11597 (CETP), and P14780 (MMP9) were up-regulated in the early sarcopenia group, while the expression levels of the other 6 proteins were down-regulated (Fig. 2B). Moreover, P11597 (CETP) and P02652 (APOA2) were involved in multiple biological processes (Table S1), suggested that these two proteins played an important role in the progression of sarcopenia and had potential diagnostic value. However, this needs to be confirmed in future studies.

## Discussion

In this study, DIA-MS was used to investigate the characteristics of serum proteome in patients with early sarcopenia, and two biomarkers with diagnostic value were identified.

Previous studies have shown that age-related loss of muscle mass affected muscle strength and function, thus weakening the mobility of the elderly [31]. Timely and effective assessment of the physical function of the early sarcopenia patients could improve their life quality. Due to the diversity of diagnostic criteria, racial and ethnic differences, the prevalence of sarcopenia between 60 and 70 years old was approximately 5–13%, and the prevalence over 80 years old was approximately 11–50% [32, 33]. Failure to diagnose accurately was a barrier to further treatment. In the past 10 years, significant progress has been made in the discovery of novel biomarkers through serum proteomic techniques. For example, Lin et al. used UPLC-tandem MS to establish the serum proteomic profile of frail older adults and suggested that serum ANGT, KG and AT levels might be effective markers for monitoring the disease progression of frail older adults [34]. Zhang et al. analyzed the serum protein expression characteristics of elderly patients with early postoperative cognitive dysfunction and suggested that fibrinopeptide A could be used as a potential biomarker [35]. These evidences demonstrated the feasibility of serum proteomic technology for screening disease biomarkers in the older adults. But the application of this technique in sarcopenia diagnostic screening has not been fully developed. In our present study, we used DIA-MS to analyze the differential characteristics of serum protein expression profile between healthy and early sarcopenia older adults. A total of 114 disease associated DEPs were identified in sarcopenia group, including 48 up-regulated protients and 66 down-regulated protients. we found that the DEPs between two groups were mainly enriched in several biological processes, such as low-density lipoprotein particle remodeling, negative regulation of transporter activity, negative regulation of immune response, and negative regulation of cytokine production. Among 114 DEPs, we further identified two proteins of diagnostic value, which were CETP and APOA2.

Our study suggested that the progression of sarcopenia might be associated with changes in serum lipoprotein particle levels, but the causal relationship between the two was still unclear. Recent studies have shown that the loss of muscle mass might be associated with dyslipidemia [36]. For example, an increase in muscle area significantly reduced the risk of hypertriglyceridemia [37]. Compared with the normal population, elderly men and women with sarcopenic obesity had a 2.5 and 1.5 higher risk of hypertriglyceridemia, respectively [38]. In this study, the level of CETP was up-regulated in early sarcopenia OAs group. CETP has been reported to regulate lipoprotein metabolism by transferring triglycerides and cholesterol esters between lipoproteins [39]. The role of CETP in the risk indication of some diseases was increasingly emphasized. For example, high level of CETP was associated with a variety of cardiovascular diseases, and reducing the level of plasma CETP could increase the level of high-density lipoprotein cholesterol, so as to effectively reduce the risk of atherosclerotic cardiovascular diseases [40]. CETP was also involved in dyslipidemia-related susceptibility to cognitive decline [41]. In contrast to CETP, the level of APOA2 was down-regulated in early sarcopenia OAs group. APOA2 was one of the main apolipoproteins of high density lipoprotein, which played a key role in lipid metabolism and obesity [42]. Previous studies have suggested that APOA2 might serve as a biomarker for diseases such as Alzheimer’s disease [43] and pancreatic cancer [44]. At present, APOA2 mainly played an auxiliary role in the diagnosis of some diseases, but its pathophysiological role has not been clarified. In addition, the diagnostic value of CETP and APOA2 in sarcopenia and whether they could be used as therapeutic targets need further research.

However, our study also had some limitations. First of all, the sample size was small and the results were not validated in a large sample size cohort. Furthermore, the nature of the cross-sectional study was unable to determine a causal relationship between APOA2, CETP and sarcopenia. However, this study also had merit, it was the first study on the serum proteome of sarcopenia patients, and we believed it provided a valuable data for patient diagnosis.

## Conclusions

In conclusion, our study provided a serum proteomic profile of sarcopenia patients, and identified two proteins (CETP and APOA2) with diagnostic value. In future studies, we will focus on the clinical diagnostic value of these two biomarkers.

## Supplementary Information

**Additional file 1: Table S1.** Results of functional enrichment analysis.

## Data Availability

The datasets generated and analysed in the current study are available in iProX website (https://www.iprox.cn//page/project.html?id=IPX0004531000).

## Abbreviations

APOA2: Apolipoprotein A2
ASM: Appendicular skeletal muscle mass
CC: Cellular component
CETP: Cholesteryl ester transfer protein
CV: Coefficient of variation
CRISP3: Cysteine-rich secretory protein 3
DDA: Data-dependent acquisition
DIA: Data-independent acquisition
DEPs: Differentially expressed proteins
KEGG: Kyoto Encyclopedia of Genes and Genomes
LPA: Lysophosphatidic acid
MS: Mass spectrometry
MMP-9: Matrix metalloproteinase-9
MF: Molecular function
PTGDS: Prostaglandin D2 synthase
PPI: Protein-protein interaction
QC: Quality control
SMI: Skeletal muscle index
AMBP: α-1-microglobulin/bikunin precursor
